# Changes in Outer Retinal Microstructures during Six Month Period in Eyes with Acute Zonal Occult Outer Retinopathy-Complex

**DOI:** 10.1371/journal.pone.0110592

**Published:** 2014-10-30

**Authors:** Yoshitsugu Matsui, Hisashi Matsubara, Shinji Ueno, Yasuki Ito, Hiroko Terasaki, Mineo Kondo

**Affiliations:** 1 Department of Ophthalmology, Mie University Graduate School of Medicine, Tsu, Japan; 2 Department of Ophthalmology, Nagoya University Graduate School of Medicine, Nagoya, Japan; Dalhousie University, Canada

## Abstract

**Purpose:**

To study the changes in the outer retinal microstructures during a six month period after the onset of acute zonal occult outer retinopathy (AZOOR)-complex by spectral-domain optical coherence tomography (SD-OCT).

**Methods:**

Seventeen eyes of 17 patients with the AZOOR-complex were studied. The integrity of the external limiting membrane (ELM), ellipsoid zone (EZ; also called the inner/outer segment junction), and interdigitation zone (IDZ; also called the cone outer segment tips) were evaluated in the SD-OCT images obtained at the initial visit and at six months. The three highly reflective bands were divided into three types; continuous, discontinuous, and absent. The integrity of the outer nuclear layer (ONL) was also assessed.

**Results:**

Among the three highly reflective bands, the IDZ was most altered at the initial visit and least recovered at six months. Fifteen of 17 eyes (88%) had a recovery of at least one of the three bands at six months in the retinal area where the ONL was intact, and these areas showed an improvement of visual field. Three eyes (18%) had retinal areas where the ONL was absent at the initial visit, and there was no recovery in both the retinal structures and visual fields in these areas.

**Conclusions:**

Our results indicate that more than 85% eyes with AZOOR-complex show some recovery in the microstructures of the outer retina during a six month period if the ONL is intact. We conclude that SD-OCT is a useful method to monitor the changes of the outer retinal microstructure in eyes with the AZOOR-complex.

## Introduction

Acute zonal occult outer retinopathy (AZOOR) is a retinal disease that was first reported by Gass [1]. AZOOR is characterized by an acute loss of one or more zones of outer retinal function, photopsia, minimal funduscopic changes, and electroretinographic (ERG) abnormalities [1]–[4]. AZOOR occurs predominantly in young women, and some patients have a viral-like illness before the onset [1], [4], [5]. The exact pathogenesis of AZOOR is still uncertain, but two possible hypotheses have been advanced; virus infection of the photoreceptors [6] and common genetic hypothesis of autoimmune/inflammatory disease [7].

In 2003, Gass suggested that retinal diseases similar to AZOOR, e.g., multiple evanescent white dot syndrome (MEWDS), multifocal choroiditis and panuveitis (MFP), punctate inner choroidopathy (PIC), acute idiopathic blind spot enlargement (AIBSE), acute macular neuroretinopathy (AMN), and AZOOR, were part of a spectrum of a single disease with similar clinical signs, symptoms, and ophthalmological findings [6]. He recommended that these should be placed in a single clinical entity called the AZOOR-complex. There are several studies that reported that two of these diseases can occur in the same patient at the same time or at different times [8]–[12].

Optical coherence tomography (OCT) is a useful method to detect subtle morphological changes in retinas affected by various pathological conditions. Past studies have demonstrated the diagnostic value of time-domain (TD) and spectral-domain (SD) OCT in eyes with AZOOR-complex. The findings showed that the integrity of the external limiting membrane (ELM), ellipsoid zone (EZ; originally called the inner/outer segment junction [13]) and/or interdigitation zone (IDZ; also called the cone outer segment tips [14]) were disrupted at the retinal areas of visual field defects in eyes with the AZOOR-complex [15]–[24]. It was also reported that the abnormalities in the outer retinal microstructures can recover during the follow-up period in some patients with the AZOOR-complex [18]–[20], [22]. However, there has not a study that analyzed how these three highly reflective bands change with time during a fixed time period for many patients.

Therefore, the purpose of this study was to determine by SD-OCT the changes in the outer retinal microstructures during a six month period after the initial visit in eyes with AZOOR-complex.

## Subjects and Methods

### Subjects

We reviewed the medical records of patients who were diagnosed with AZOOR-complex who visited the Mie University Hospital or the Nagoya University Hospital from September 2007 to June 2013. Because the purpose of this study was to determine the changes of the retinal microstructures at the early stages of AZOOR-complex, only the patients whose initial examination was ≤3 months from the onset were studied. In addition, only the patients who were followed for at least six months after the initial visit were included. Based on these criteria, seventeen eyes of 17 patients with the AZOOR-complex were studied.

All patients had undergone a complete eye examination including best-corrected visual acuity (BCVA) measured by a standard Japanese decimal visual acuity chart at 5 m, slit-lamp biomicroscopy, color fundus photography, Humphrey static perimetry (30-2 program), and SD-OCT. Fluorescein angiography was performed only at the initial visit. Full-field electroretinograms (ERGs) or multifocal ERGs were recorded at the initial visit for a correct diagnosis of the AZOOR-complex [2], [3].

The procedures used conformed to the tenets of the World Medical Association's Declaration of Helsinki. Mie University Institutional Ethics Review Board approved this retrospective study of the patients' medical records. Written informed consent was not given by participants for their clinical records to be used in this study, but patient information was anonymized and de-identified prior to analysis.

### Spectral-domain optical coherence tomography (SD-OCT)

All of the patients had undergone SD-OCT examinations with the Spectralis OCT (HRA+OCT, Heidelberg Engineering) or the Cirrus HD-OCT (version 5.1, Carl Zeiss Meditec). Following the dilation of the pupils, the retinal tomographic images of 9 mm (approximately 30°) horizontal scans for Spectralis or horizontal 6 mm scans for Cirrus were obtained across the fovea. Depending on the image quality, B-scans were averaged.

We evaluated the SD-OCT findings at the initial visit and at six months. The reason why we selected the SD-OCT findings at 6 months was because Gass et al. [4] had reported that the alterations of the patients' vision stabilized within six months of the onset in 77% of the patients. We evaluated the integrity of the three highly reflective bands at the outer retina obtained by the SD-OCT; the external limiting membrane (ELM), ellipsoid zone (EZ) [13], and interdigitation zone (IDZ) [14]. The integrity of these bands was divided into three types; “continuous”, “discontinuous”, or “absent”. The bands were defined as “continuous” when they were seen clearly and appeared to be continuous. The bands were defined as being “discontinuous” when they were blurred or interrupted. The bands were defined as “absent” when they were not identified at the area of the visual field defect. These decisions were made by two retinal specialists (YM and HM) independently and were masked to the other clinical findings. In addition, the preservation of outer nuclear layer (ONL) was also assessed.

To evaluate the changes in the outer retinal highly reflective bands more quantitatively, a longitudinal reflectivity profile (LRP) was created by previously described methods [13], [25], [26]. In brief, one vertical straight line was drawn at the retinal area of the visual field defect. The LRPs were made by calculating median values of pixels across each level of 20 adjacent A-scans using ImageJ (National Institutes of Health, Bethesda, MA; available at rsbweb.nih.gov/ij/download.html).

## Results

The clinical characteristics and SD-OCT findings of the 17 Japanese patients with AZOOR-complex (three men and 14 women; age, 19–49 years) are summarized in Table 1. The diagnosis of our 17 patients included 10 with AZOOR, 4 with MEWDS, and 3 with AIBSE. The average interval between the onset of symptoms and examination in our hospital was 3.6 weeks with a range of 1 to 12 weeks. The average spherical equivalent refractive error was -4.4 diopters (D) with a range of -0.5 to -13.5 D. The type of visual field defects included two with a central scotoma, six with a paracentral scotoma, two with a temporal scotoma, and seven with a centro-temporal scotoma (Table 1).

**Table 1:** Clinical Characteristics and SD-OCT findings of Patients with AZOOR-Complex.

Case/Sex/Age (y)/Eye	Diagnosis	Initial visit from the onset (week)	Spherical equivalent refractive error (dioptor)	Type of Scotoma	BCVA initial/6M	ELM initial/6M	EZ initial/6M	IDZ initial/6M	Improvement in visual field
1/F/30/Right	AZOOR	2	−0.5	Paracentral	1.0/1.2	II/I	II/I	III/II	Significant
2/M/35/Right	AZOOR	4	−0.5	Paracentral	1.2/1.5	I/I	I/I	II/I	Significant
3/F/34/Right	AZOOR	1	−11.5	Centro-temporal	0.5/0.7	II/II	III/III	III/III	None
4/F/19/Right	AZOOR	2	−3.0	Temporal	0.9/1.2	II/I	II/I	III/III	Mild
5/F/49/Left	AZOOR	12	−13.5	Centro-temporal	0.8/0.6	III/III	III/III	III/III	None
6/F/30/Right	AZOOR	4	−7.0	Centro-temporal	0.5/0.8	I/I	II/I	II/III	Mild
7/F/37/Left	AZOOR	1	−8.5	Centro-temporal	0.6/1.0	II/I	II/I	III/III	Mild
8/F/25/Left	AZOOR	8	−6.6	Centro-temporal	0.2/0.8	I/I	II/I	III/III	Mild
9/M/42/Right	AZOOR	2	−6.0	Paracentral	1.2/1.2	I/I	II/I	III/II	Mild
10/F/19/Right	AZOOR	1	−4.5	Centro-temporal	0.6/1.2	II/II	II/I	III/II	Significant
11/F/35/Right	MEWDS	2	−10.0	Central	0.5/1.2	II/I	III/I	III/II	Significant
12/F/31/Left	MEWDS	1	−6.5	Central	0.3/1.2	II/I	II/I	III/I	Significant
13/M/39/Right	MEWDS	8	−0.5	Centro-temporal	0.2/1.2	II/I	III/I	III/II	Significant
14/F/24/Right	MEWDS	1	−2.75	Paracentral	0.8/1.2	II/I	II/I	III/II	Significant
15/F/47/Right	AIBSE	1	−2.0	Temporal	1.2/1.2	I/I	II/I	III/III	None
16/F/37/Left	AIBES	3	−0.5	Paracentral	1.2/1.0	II/I	II/I	III/I	Significant
17/F/29/Left	AIBSE	8	−5.75	Paracentral	1.0/1.2	I/I	II/I	III/II	Significant

Abbreviations: AZOOR, acute zonal occult outer retinopathy; MEWDS, multiple evanescent white dot syndrome; AIBSE, acute idiopathic blind spot enlargement; BCVA, best-corrected visual acuity; ELM, external limiting membrane; EZ, ellipsoid zone; IDZ, interdigitation zone; I, continuous; II, discontinuous; III, absent.

### Case Presentations

#### Case 11: MEWDS Associated with ONL Loss

**Figure 1:**
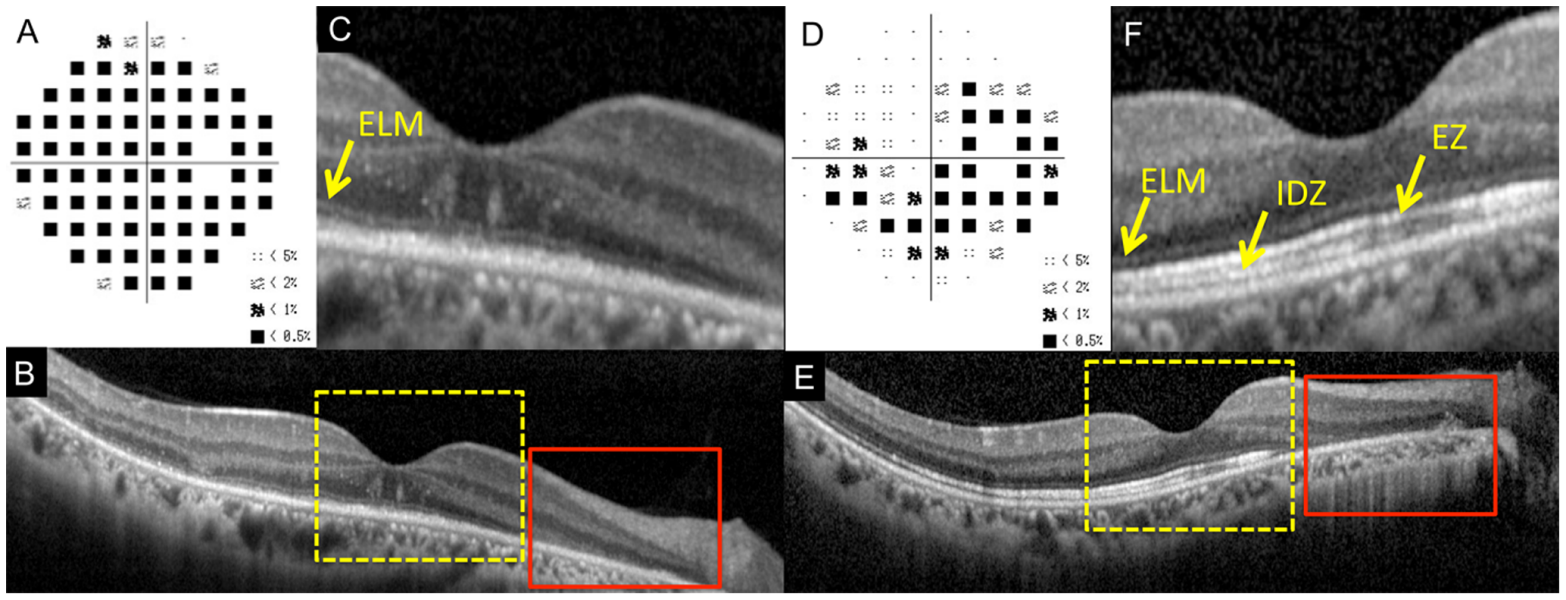
Static visual field and spectral-domain optical coherence tomographic (SD-OCT) results of the right eye of Case 11 at the initial visit (A–C) and six months after the initial visit (D–F). A: Deviation plot obtained by the Humphrey 30-2 program at the initial visit. B: Horizontal SD-OCT image through the fovea at the initial visit. C: Magnified view of the area outlined by dashed yellow line box in the image of B. D: Deviation plot obtained with the Humphrey 30-2 program at six months after the initial visit. E: Horizontal SD-OCT image through the fovea at six months after the initial visit. F: Magnified view of the area outlined by dashed yellow line box in the image of E. ELM, external limiting membrane. EZ, ellipsoid zone. IDZ, interdigitation zone. This case had the retinal area where the outer nuclear layer (ONL) was completely absent (red line boxes).

A 35-year-old myopic woman presented with complaints of acute vision reduction and photopsia in her right eye. Her decimal best-corrected visual acuity (BCVA) was 0.5 OD, and perimetry showed a visual field defect within 30 degrees of the fovea (Fig. 1A). Fundus examination showed multiple small, gray-white patches at the level of RPE and outer retina in the mid-peripheral region, and these white patches disappeared within four weeks without treatment. Based on these clinical findings, she was diagnosed with MEWDS. She was followed up without any treatments.

Her SD-OCT findings at the initial visit are shown in Figures 1B and 1C. In the central retinal area where the ONL was intact (yellow dotted square, Fig. 1B), the ELM was judged to be “discontinuous”, and the EZ and IDZ were classified as being “absent”. This was because these two lines were merged with the RPE-Bruch's membrane complex and were not identified as independent bands (Fig. 1C).

At six months after the initial visit, the decimal BCVA had improved to 1.2, and the visual field had recovered at many points (Fig. 1D). SD-OCT also showed an improvement in the outer retinal microstructures but only at the area of the intact ONL (yellow dotted square, Figs. 1E and 1F). At this time, the ELM and EZ were judged as “continuous”, but the IDZ was still judged as “discontinuous” at the central area.

We also noted that this patient had an area of the retina where the ONL was completely absent at the initial visit (red square, Fig. 1B), and there was no recovery of the both the visual field and the SD-OCT image in this area at 6 months (red square, Fig. 1E).

#### Case 1: AZOOR with Recovery of Outer Retinal Microstructures

**Figure 2:**
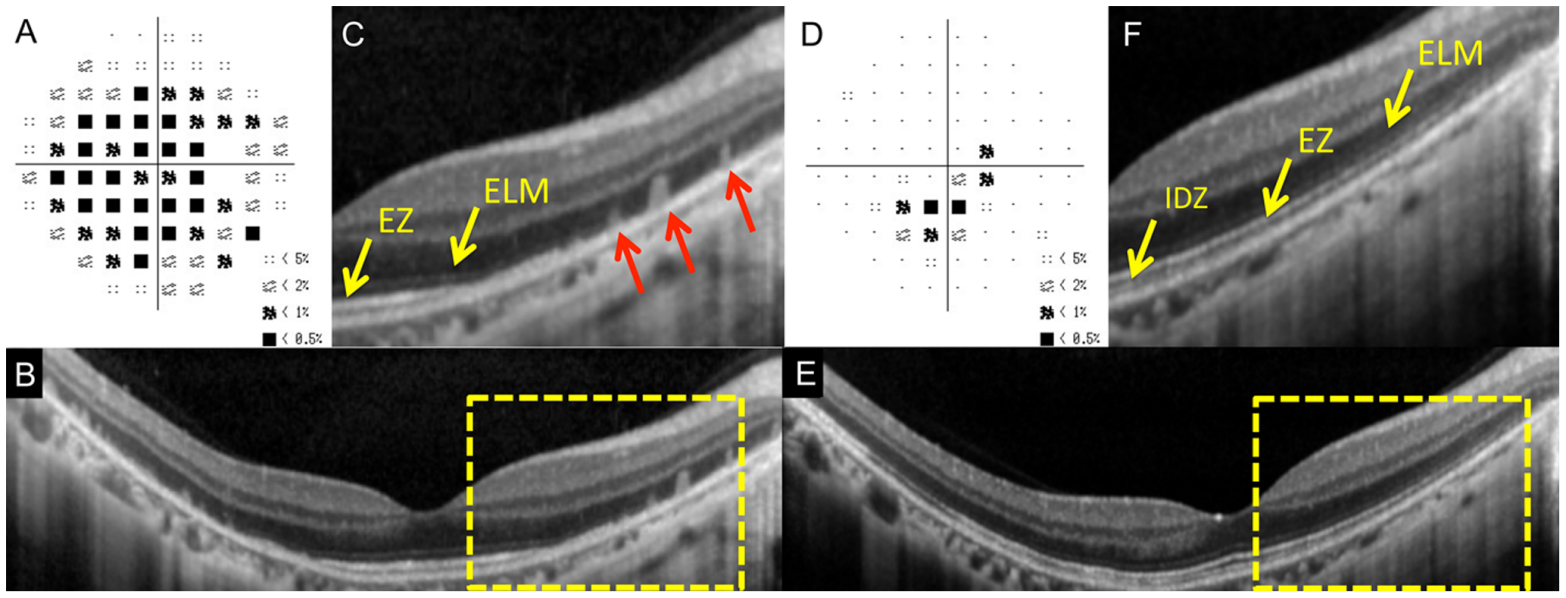
Static visual field and spectral-domain optical coherence tomography (SD-OCT) results of the right eye of Case 1 at the initial visit (A–C) and six months after the initial visit (D–F). A: Deviation plot obtained with the Humphrey 30-2 program at the initial visit. B: Horizontal SD-OCT image through the fovea at the initial visit. C: Magnified view of the area outlined by dashed yellow line box in B. D: Deviation plot obtained with the Humphrey 30-2 program at six months after the initial visit. E: Horizontal SD-OCT image through the fovea at six months after the initial visit. F: Magnified view of the area outlined by dashed yellow line box in the image of B. ELM, external limiting membrane. EZ, ellipsoid zone. IDZ, interdigitation zone. Several column-shaped highly reflective materials are seen at the outer retinal area of visual field defect at the initial visit (red arrows).

A 30-year-old emmetropic woman had an acute onset of photopsia and vision reduction in her right eye. Her decimal BCVA was 1.0 OD, and fundus examination and fluorescein angiography were essentially normal. However, perimetry showed severely decreased retinal sensitivities within 20 degrees of the fovea in the right eye (Fig. 2A). Multifocal ERGs also showed reduced focal ERGs within 20 degrees of the fovea. Based on these findings, this patient was diagnosed with AZOOR. She was followed without any treatments.

Her SD-OCT findings at the initial visit are shown in Figures 2B and 2C. The ELM and EZ were judged to be “discontinuous”, because they were disrupted away from the macula (Figs. 2B and 2C). The IDZ was judged to be “absent” because this band was not identified over the entire 9 mm scan. Interestingly, the retina had highly reflective materials in columns which passed through the ONL from the RPE at the area of visual field defect (red arrows, Fig. 2C). Similar highly reflective materials have been reported in a patient with MEWDS [20].

After six months, there was a marked improvement in her visual fields (Fig. 2D), and SD-OCT showed a recovery of the outer retinal microstructures. At this time, the ELM and EZ were judged to be “continuous”. However, the IDZ was still “discontinuous”, because it was only identified in the central area (Figs. 2E and 2F). We also noticed that the columnar highly reflective materials were not present at six months. The ONL was preserved in the initial and 6 months SD-OCT images.

#### Case 2: AZOOR with Intact EZ at Initial Visit

**Figure 3:**
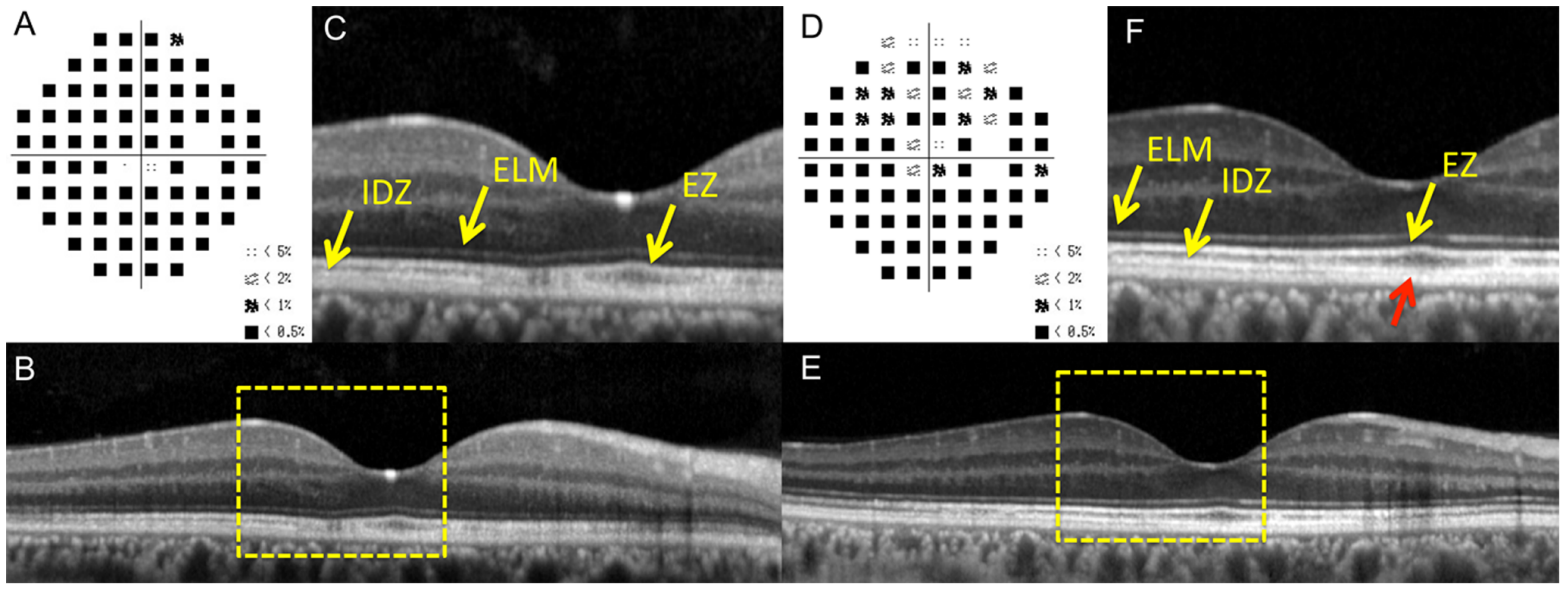
Static visual field and spectral-domain optical coherence tomographic (SD-OCT) results in the right eye of Case 2 at the initial visit (A–C) and six months after the initial visit (D–F). A: Deviation plot obtained with the Humphrey 30-2 program at the initial visit. B: Horizontal SD-OCT image through the fovea at the initial visit. C: Magnified view of the area outlined by dashed yellow line box in the image of B. D: Deviation plot obtained by the Humphrey 30-2 program at six months after the initial visit. E: Horizontal SD-OCT image through the fovea at six months after the initial visit. F: Magnified view of the area outlined by dashed yellow line box in the image of B. The COST line is still blurred near the fovea (red arrow). ELM, external limiting membrane. EZ, ellipsoid zone. IDZ, interdigitation zone.

A 35-year-old healthy emmetropic man reported that he had an acute paracentral visual field depression and photopsia in his right eye. His decimal BCVA was 1.2 OD. His fundus and fluorescein angiography were normal, but visual field showed extensive defects outside the fovea in the right eye (Fig. 3A). His scotopic and photopic full-field ERGs were reduced but only in the right eye. Based on these findings, he was diagnosed with AZOOR. He was treated with intravenous drip methylprednisolone.

His SD-OCT findings at the initial visit are shown in Figures 3B and 3C. The ELM and EZ were judged to be “continuous”, but the IDZ was judged to be “discontinuous” (Fig. 3B & 3C).

Six months later, he reported some improvements of his visual symptoms, and his visual field showed recovery at several points (Fig. 3D). At this time, the ELM and EZ were judged to be “continuous”, but the IDZ was judged to be “discontinuous”, because it was still blurred near the fovea (yellow arrow, Fig. 3F, red arrow). The ONL was intact both at the initial visit and at 6 months.

**Figure 4:**
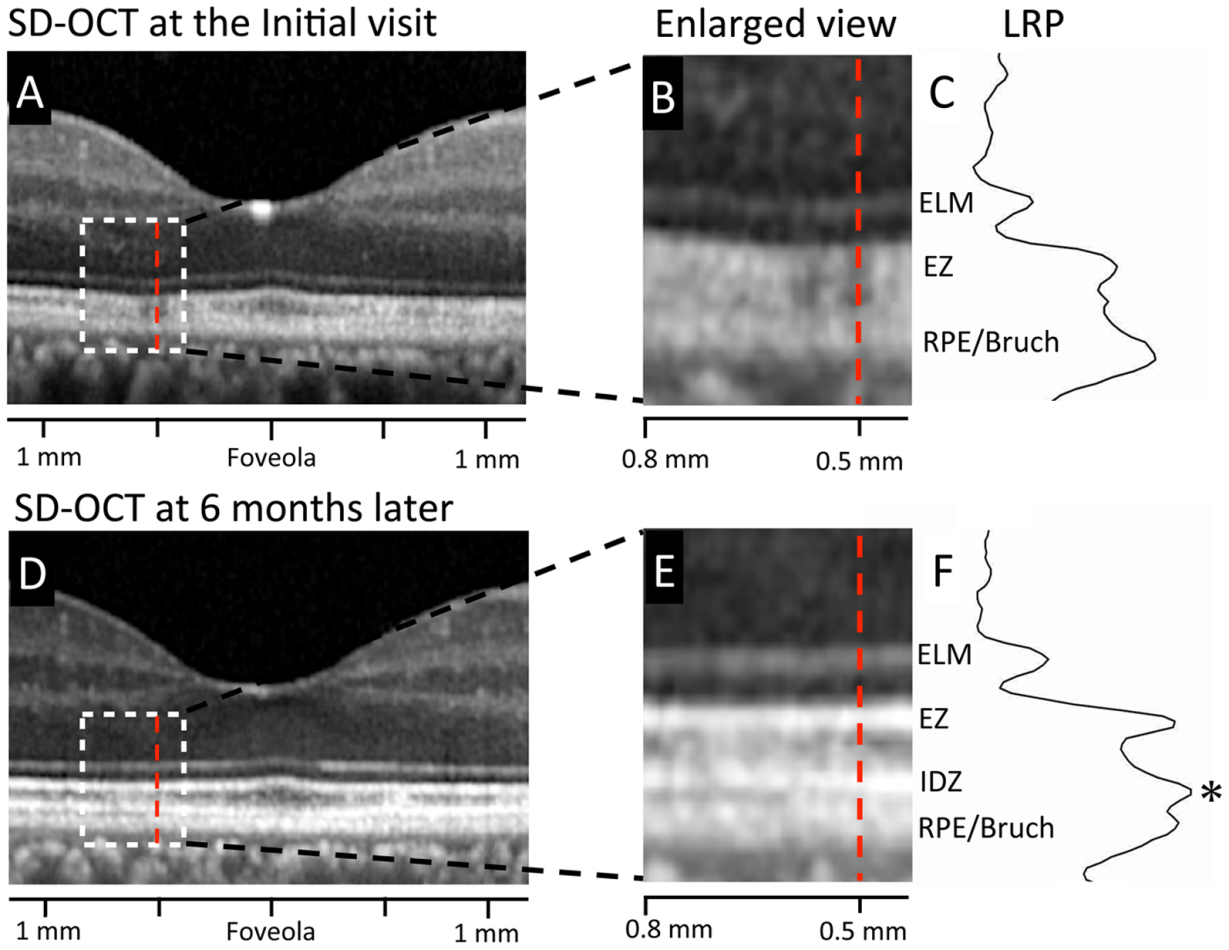
Results of longitudinal reflectivity profile (LRP) in the retina of Case 2. A. Horizontal SD-OCT scan through the fovea at the initial visit. B. Magnified view of the area outlined by dashed white line box in the image of A. C. Longitudinal reflectivity profile (LRP) along the vertical line at 0.5 mm temporal from the foveola (red dotted line) at the initial visit. D. Horizontal SD-OCT scan through the fovea at six months after the initial visit. E. Magnified view of the area outlined by dashed white line box in the image of D at six months after the initial visit. F. Longitudinal reflectivity profile (LRP) along the vertical line at 0.5 mm temporal from the foveola (red dotted line) at six months after the initial visit. At the retinal area of 0.5 mm temporal from the foveola, IDZ was nearly undetectable at the initial visit, but the peak of IDZ was clearly detectable as a third highly reflective band at six month later (asterisk). ELM, external limiting membrane. EZ, ellipsoid zone. IDZ, interdigitation zone. RPE/Bruch, retinal pigment epithelium/Bruch's membrane complex. LRP, longitudinal reflectivity profile.

To evaluate the changes in outer retinal high-reflective bands more quantitatively, a longitudinal reflectivity profile (LRP) was created in the retina of Case 2 (Fig. 4). One vertical straight line was drawn at 0.5 mm temporal retina from the foveola (red dotted lines of Fig. 4). We found that that IDZ was undetectable at the initial visit, but it became detectable as a third highly reflective band six month later (Figs. 4C and 4E).

#### Case 3: AZOOR with Worsening of Visual Fields

**Figure 5:**
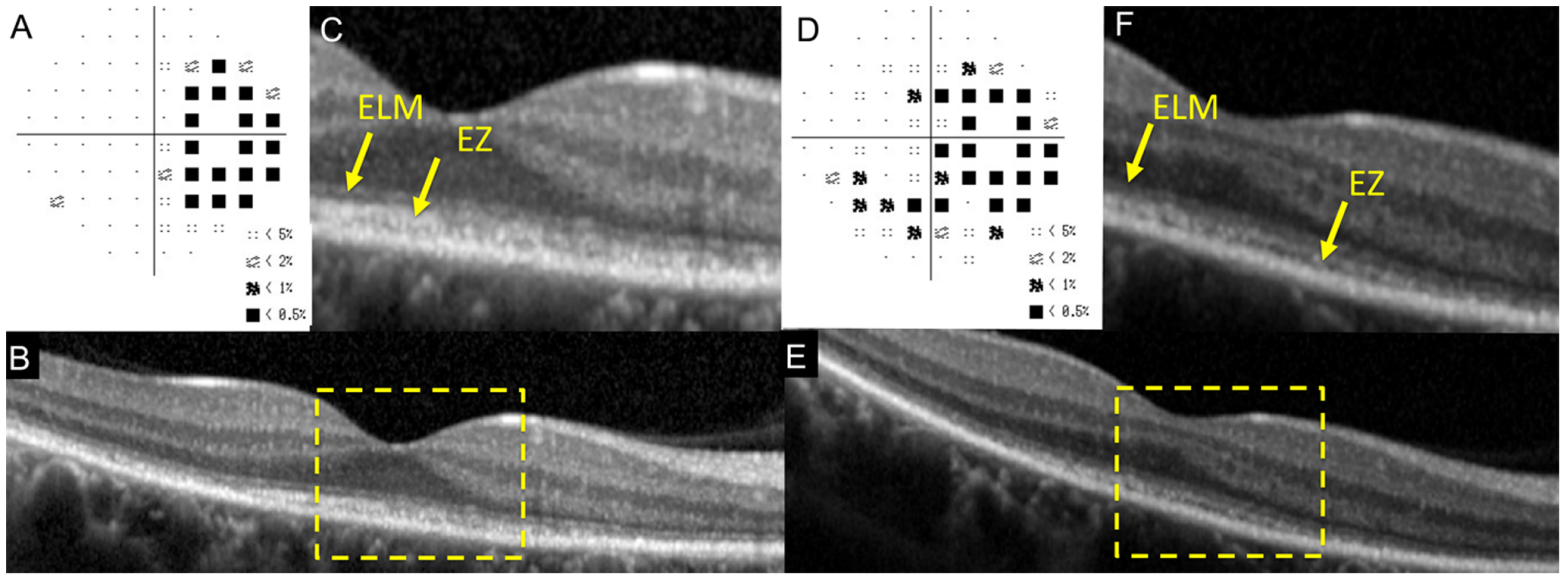
Static visual field and spectral-domain optical coherence tomographic (SD-OCT) results in the right eye of Case 3 at the initial visit (A–C) and six months after the initial visit (D–F). A: Deviation plot obtained by the Humphrey 30-2 program at the initial visit. B: Horizontal SD-OCT image through the fovea at the initial visit. C: Magnified view of the area outlined by dashed yellow line box in the image of B. D: Deviation plot obtained by the Humphrey 30-2 program at six months after the initial visit. E: Horizontal SD-OCT image through the fovea at six months after the initial visit. F: Magnified view of the area outlined by dashed yellow line box in the image of E. ELM, external limiting membrane. EZ, ellipsoid zone. IDZ, interdigitation zone.

A 34-year-old myopic woman reported experiencing photophobia and vision reduction of one month duration in her right eye. She had a history of Basedow disease for eight years. Her decimal BCVA was 0.5 OD. Fundus examination and fluorescein angiography were normal, but Humphrey visual field tests revealed a temporal scotoma extending into the fixation point in the right eye (Fig. 5A). The multifocal ERGs were reduced in the centro-temporal field. Based on these clinical findings, she was diagnosed with AZOOR. She was treated with intravenous drip methylprednisolone.

Her SD-OCT findings of the right eye at the initial visit are shown in Figures 5B and 5C. Her ELM and EZ were judged to be “discontinuous”, and the IDZ was judged to be “absent”. Despite intravenous drip methylprednisolone and following orally administrated predonisolone for six months, she felt that there was a gradual worsening of her visual decrease, and visual field tests showed an enlargement of the temporal scotoma (Fig. 5D). At this time, the ELM and EZ were judged to be “discontinuous”, and the IDZ still remained “absent” (Figs. 5E and 5F). The ONL thickness was also reduced at the areas of the visual field defects.

### Summary of SD-OCT findings

**Figure 6:**
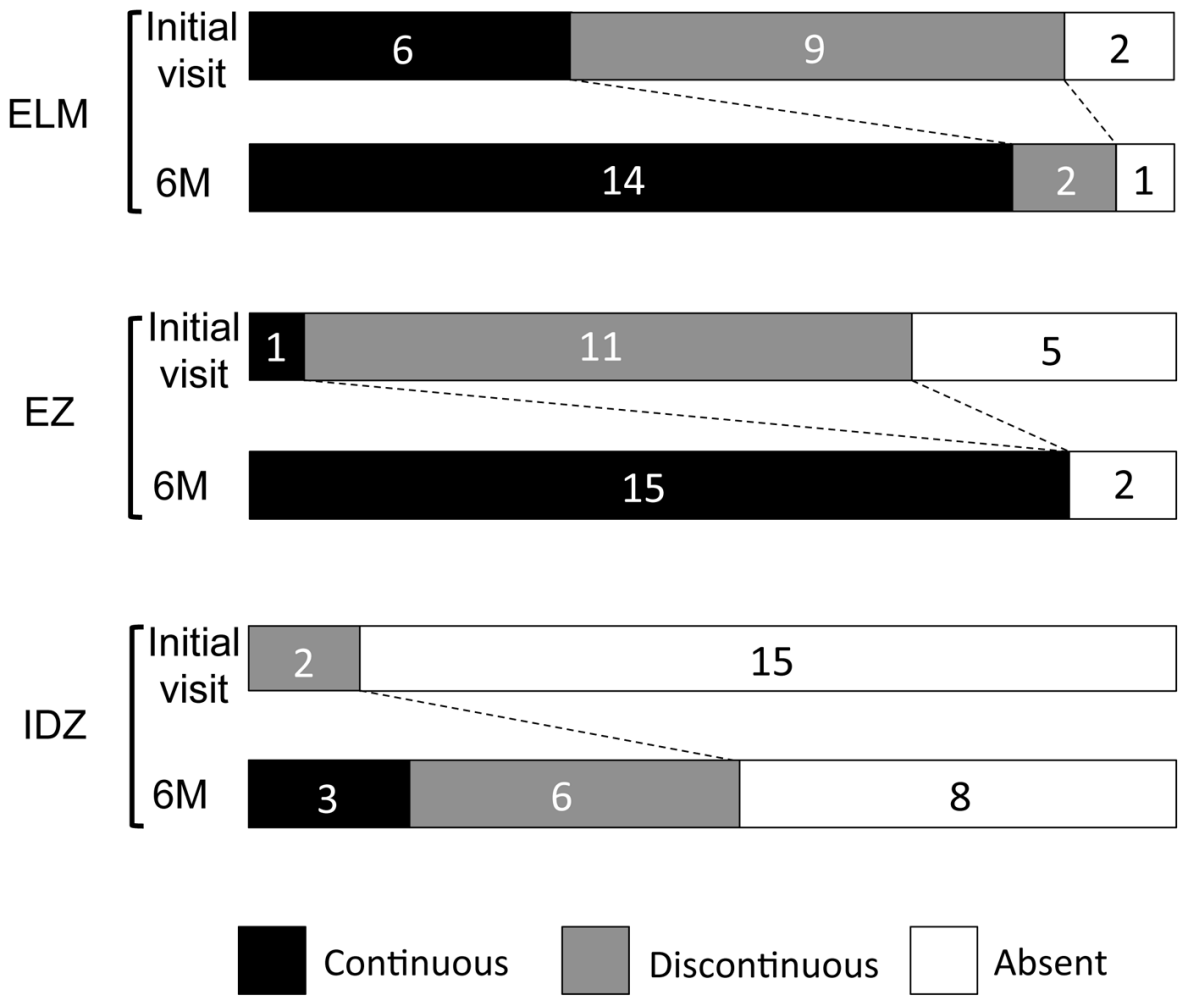
Summary of findings of the three highly reflective bands at the outer retina obtained by SD-OCT at the initial visit and six months later. These three lines were divided into three types; “continuous”, “discontinuous” and “absent”. ELM, external limiting membrane. EZ, ellipsoid zone. IDZ, interdigitation zone.

The changes in the ELM, EZ, and IDZ at the initial visit and six months later are summarized in Figure 6 (see also Table 1). The status of the three highly reflective bands were evaluated at the retinal areas with visual field defects. In this evaluation, we excluded the retinal areas of undetectable ONL, because we noted that the retinal areas with loss of ONL at the initial visit did not show any improvement both in the SD-OCT findings and the visual field defects. Therefore in Figure 6, the results are shown only at the retinal areas of intact ONL.

We found that the IDZ was most vulnerable among the three outer retinal bands at the initial visit. There were no AZOOR-complex patients who had a continuous IDZ at the area of visual field defect at the initial visit. The IDZ was also most slow to recover at six months after the initial visit. Even at six months, the IDZ was continuous only in three eyes (18%), discontinuous in six eyes (35%), and still absent in eight eyes (47%).

When compared to the IDZ, the ELM and EZ were relatively well preserved at six months. The ELM was continuous in 14 of 17 eyes (82%) at six months. Similarly, the EZ was continuous in 15 of 17 eyes (88%) at six months (Fig. 5).

We also found that 15 of 17 eyes (88.3%) had a recovery of at least one of the three bands during six months (Table 1) if the ONL was intact, and these 15 eyes also showed an improvement of the visual field defects.

## Discussion

We investigated the changes in the outer retinal microstructures at the initial visit and six months later in 17 eyes with AZOOR-complex using the SD-OCT. There have been many case series which described the change of OCT findings with time in AZOOR-complex patients [12], [15]–[24], but the best of our knowledge, this is the first systematic report focusing on the changes in the three outer retinal bands of SD-OCT during a fixed time period.

We found that the IDZ was most vulnerable among the three outer retinal bands in the retina of AZOOR-complex. At the initial visit, the IDZ was abnormal, i.e., discontinuous or absent, in all 17 eyes (100%), while the ELM and EZ were abnormal in 11 (65%) and 16 eyes (94%), respectively (Fig. 6). Similarly, at six months after the initial visit, the IDZ was still abnormal in 14 eyes (82%), while the ELM and EZ were abnormal in only three (18%) and two eyes (12%), respectively. These results indicate that the IDZ is the most vulnerable microstructure and can be used to detect and follow the alterations of the outer retina in eyes with AZOOR-complex.

The origin of IDZ has not been established, and is currently thought to correspond to the junction [14] or contact cylinder [13] between the RPE apical processes and the external portion of the cones. Thus, this band is thought to be a useful indicator of the integrity of outer segments of the photoreceptors. Recent studies have reported that the integrity of the IDZ was significantly correlated with visual function in different retinal diseases including occult macular dystrophy [27], [28], epiretinal membrane [29]–[31], and central serous chorioretinopathy [32]. On the other hand, it is also known that the IDZ cannot be identified clearly even in some normal subjects [33], [34]. Thus, Rii et al. reported that the incidence of eyes with an intact foveal IDZ was about 95% in normal individuals [33]. Taken together, we now interpret our findings by concluding that the IDZ is the most vulnerable microstructure in eyes with our AZOOR-complex patients. This is because the visibility of the IDZ is most easily affected when the photoreceptors are damaged and not necessarily because this region is the primary site of this disorder.

There are several reports suggesting that the abnormality of EZ was present in the region of the visual field defects in AZOOR-complex [12], [15]–[24]. Our results agree with this because most of our patients (16 of 17 eyes, 94%) had abnormal EZ at the region of the visual field defect at the initial visit. However, we also noted that one of our patients had a “continuous” EZ even at the retinal area of visual field defect at the initial visit (Case 2). In this area, only the IDZ was “discontinuous” (Fig. 3C). Tsunoda et al. [23] recently described two AZOOR patients whose EZ was normal, but the IDZ was not present or indistinct at the retinal area of visual field defect. So et al. [22] also reported an AZOOR patient whose EZ recovered, but the IDZ was still absent at the one month follow-up examination. We recommend focusing on not only the EZ but also the IDZ to enhance the detection of abnormal retinal microstructures in eyes with the AZOOR-complex [22], [23].

Spaide et al. [18] reported that there was no visual field or anatomic improvements in the retinal regions where there was outer nuclear loss and that the improvement of scotoma and restoration of EZ were only seen in areas that had no loss of outer nuclear layers. Our results agree with their findings. We noted that three of 17 eyes (18%, Case 5, 11, and 16) that had retinal regions with a loss of the ONL at the initial visit did not show any recovery both in the retinal microstructures or the visual fields at these regions during six months (red squares of Fig. 1). These results support the idea that the photoreceptor outer segments can recover by the process of renewal only when the photoreceptor cell bodies are intact [18], [20], and also suggests that the OCT findings of ONL can be useful in predicting whether the visual field defect can recover in eyes with AZOOR-complex.

Many of our patients with AZOOR-complex had myopia, and the average spherical equivalent refractive error in our 17 eyes was -4.4 D. This is consistent with past reports showing a high prevalence of myopia in eyes with AZOOR-complex [4], [35]. In this study, we also noted that the eyes with more severe myopia of>−5.0 D tended to have worse outcomes with abnormal EZ or IDZ at 6 months (Table 1). It should be interesting to study the correlation between the degree of myopia and the severity of outer retinal damage in more patients with AZOOR-complex.

There are two major limitations in this study. The first limitation is the small number of patients who were studied retrospectively. Because the AZOOR-complex is a very rare disease, we could not collect many patients from only two institutions. In addition, some patients were excluded because of insufficient SD-OCT or clinical data. Longer prospective studies with a larger number of patients may clarify more detailed information on the structural and functional changes with time in AZOOR-complex.

The second limitation is that we have combined the three different subtypes of the AZOOR-complex, MEWDS, AZOOR, and AIBSE. Although Gass et al. [6] suggested that these diseases may be part of a spectrum of a single disease, the prognosis is clearly different among the different types of AZOOR-complex [36]. Therefore, subgroup analysis for each type of disease may add more useful information.

Despite these limitations, our results demonstrated that 15 of 17 eyes (88%) with AZOOR-complex have some recovery of the retinal microstructures during six months if the ONL is intact. We also showed that the IDZ was the most vulnerable at the initial visit, and difficult to recover in this disorder. The SD-OCT was very useful for monitoring the changes of the outer retinal microstructure in eyes with the AZOOR-complex.
